# Quantitative and qualitative investigation of shunt failure in an *in vitro* hemorrhagic hydrocephalus model

**DOI:** 10.3389/fbioe.2025.1591952

**Published:** 2025-07-01

**Authors:** Shaheer Ajaz, Ahmad Faryami, Ayman Suhrawardy, Carolyn A. Harris

**Affiliations:** ^1^ Department of Biomedical Engineering, Wayne State University, Detroit, MI, United States; ^2^ Department of Chemical Engineering and Materials Science, Wayne State University, Detroit, MI, United States

**Keywords:** hemorrhage, posthemorrhagic hydrocephalus, ventricular catheter, external ventricular drain, in vitro, time-lapse, shunt obstruction, shunt failure

## Abstract

**Background:**

Previous studies on posthemorrhagic hydrocephalus (PHH) reported notable shunt failure rates as high as 45.1% within the first six months of placement. Using the Automated, *In Vitro* Model for Hydrocephalus Research (AIMS), we investigated the impact of CSF-like diluted blood solution on ventricular catheters and external ventricular drains (EVD) using quantitative (flow and pressure measurements) and qualitative (real-time brightfield video capture) methods.

**Methods:**

The shunt failure criteria were set at greater than or equal to 30 ${cmH}_{2}O$ such that the experiment was manually terminated if pressure surpassed 30 ${cmH}_{2}O$ differential pressure at any point within 24 h. To investigate our hypotheses, shunt failure was investigated in nine groups of catheters (n = 4 per group). Saline was utilized to dilute heparinized whole porcine blood to prepare blood-saline solutions at two concentrations of 0.05 and 0.5 volume percent (v/v%). The bulk input flow rate was set at 0.3 $\frac{mL}{\min}$ for all samples except where the flow rate was intentionally varied. The rate of pressure increase (pressurization rate) was used in all statistical analyses.

**Results:**

Over 500 h of data were gathered without leaks or ruptures. There was a statistically significant difference in the pressurization rate across blood concentration groups (*P = 0.005*). All the barium-impregnated and antibiotic-impregnated catheters reached 30 ${cmH}_{2}O$ threshold at 0.5% concentration. A statistically significant difference was observed in the pressurization rate (*P = 0.029*). A more pronounced difference was observed between the antibiotic-impregnated ventricular catheters and antibiotic-impregnated EVDs. All four EVDs completed the 24-hour experiment and did not reach the pressure threshold (*P = 0.029*).

**Conclusion:**

A direct relationship was observed between blood concentration and the pressurization rate, such that shunts and EVDs that were exposed to higher concentrations of blood consistently reached 30 ${cmH}_{2}O$ threshold while the samples in the low-concentration groups did not. In contrast, a reverse relationship was observed between bulk flow rate and pressurization rate, such that the samples exposed to 0.5% at 0.7 $\frac{mL}{\min}$ had a significantly smaller pressurization rate than samples exposed to 0.5% at 0.3 $\frac{mL}{\min}$ (*P = 0.029*). These findings highlight the role of clot formation in PHH shunt obstruction and suggest avenues for improved catheter design or clinical management.

## Background

Hydrocephalus is a neurological condition precipitated by an abnormal accumulation of cerebrospinal fluid (CSF) that may lead to neurologic injury or death if left untreated. It is estimated that hydrocephalus impacts between 1 and 2 out of 1000 infants in the United States alone, contributing to $2 billion in healthcare costs annually (Serlo et al., 1990; Koschnitzky et al., 2023). Hydrocephalus may arise from a multitude of etiological factors, including congenital anomalies, infectious agents, neoplastic growth, traumatic events, or Intraventricular hemorrhages (IVH) (Hinson et al., 2010; Abebe et al., 2022). IVH is one of the leading causes of brain damage and death in infants born before 37 weeks’ estimated gestitional age and is often a precursor to Posthemorrhagic hydrocephalus (PHH) (Hinson et al., 2010; Schindler et al., 2017; Naff, 1999; Christian et al., 2016; Valdez Sandoval et al., 2019).

The most common method of hydrocephalus management is the surgical insertion of a shunt system to redirect the CSF for absorption. Despite 70 years of continued efforts, shunts still fail at a shocking rate, necessitating medical intervention and revision surgeries. In pediatric patients alone, the failure rate is nearly 50% within the first two years and 85% within 10 years of shunt implantation (Peng and Rose, 2017; Enchev and Oi, 2008). While the revision rate in patients with PHH varies considerably, patients with PHH are at an even higher risk for shunt failure and have a significantly lower shunt survival time. For instance, in a previous study on adult patients with hemorrhage-related hydrocephalus, 45.1% of the shunts failed within the first six months. In another investigation on the pediatric cohort, a 13.8% five-year survival rate was reported in Patients with PHH; significantly smaller than the 42.2% five-year survival rate reported in patients with congenital hydrocephalus (Notarianni et al., 2009). Furthermore, our recent investigation reported posthemorrhagic hydrocephalus as the most common etiology (48.9% of total cases) in a cohort of 305 patients, highlighting the prevalence of posthemorrhagic hydrocephalus (Garcia-Bonilla et al., 2024). Although the exact mechanism of shunt failure is still unknown, anecdotal evidence from our patient repository of explanted failed catheters would in combination with published literature allow us to infer that shunt obstruction by coagulated blood is a common cause of shunt failure in Patients with PHH (Hinson et al., 2010; Taylor and Peter, 2001; C et al., 2017).

Previous investigations of PHH have implemented a range of *in vitro* and *in vivo* models with a common intention of studying PHH mechanisms and/or exploring new therapeutic targets and methods for PHH management (C et al., 2017; Yang et al., 2022; Devathasan et al., 2021). In this study, we sought to investigate the obstruction of ventricular catheters in a CSF-like diluted 0.05% and 0.5% blood solution with a secondary goal to qualitatively investigate the interaction of blood with ventricular catheters in real-time. The *in vitro* model interfaced catheters with pulsatile flow at clinically reported bulk flow rates and blood concentrations (Fam et al., 2017; Javinsky, 2012). We investigated the rate of failure in antibiotic-impregnated catheters, barium-impregnated catheters, and antibiotic-impregnated external ventricular drain (EVD) using quantitative (flow and pressure measurements) and qualitative (real-time brightfield footage) methods.

## Materials and methods

**FIGURE 1:**
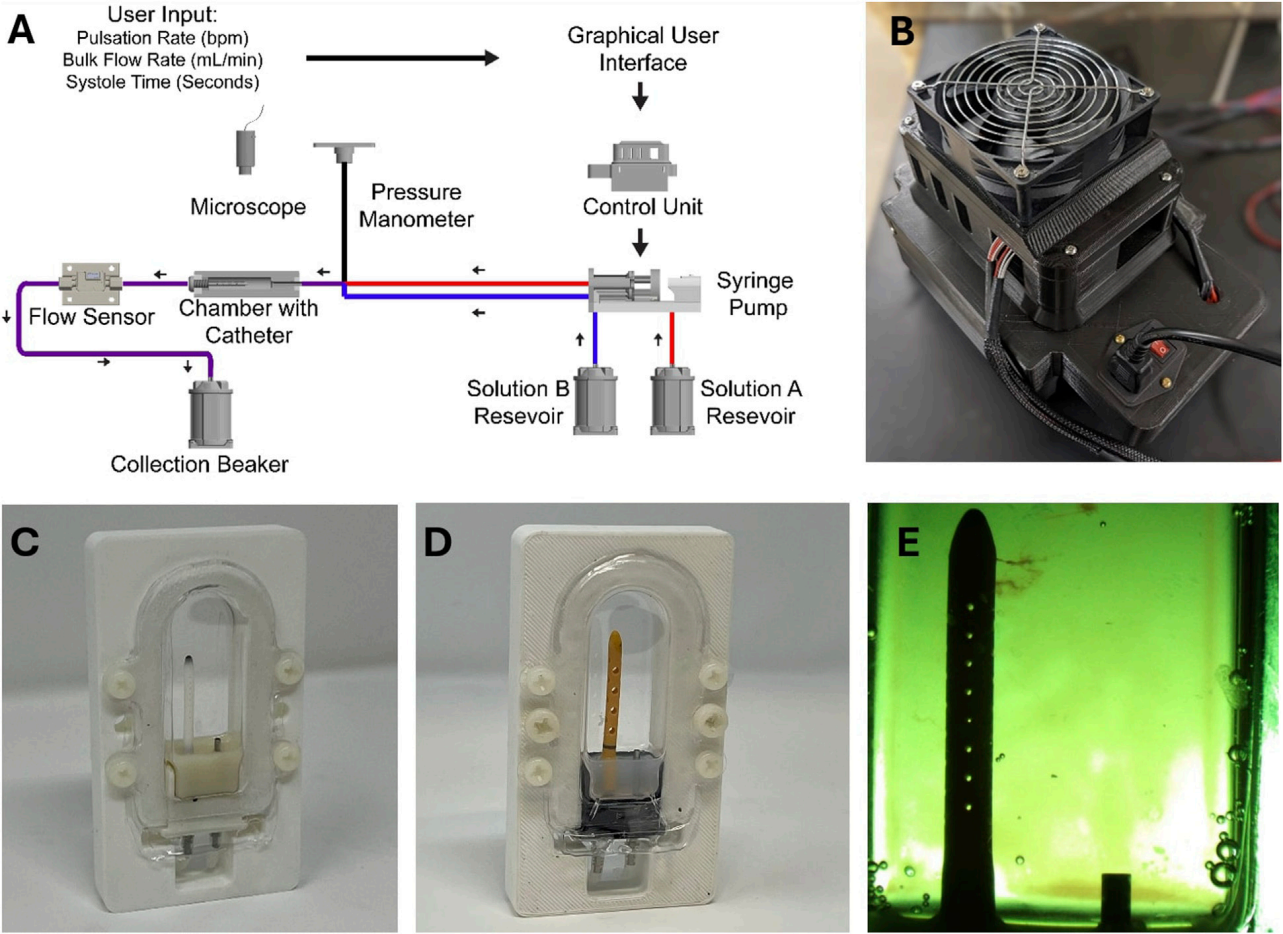
The experimental setup. **(A)** The schematic of the experimental setup. **(B)** Top view of AIMS control unit. **(C)** An example of a ventricular catheter in a PETG bioreactor chamber. **(D)** An example of an EVD in a PETG bioreactor chamber. **(E)** A top view of a ventricular catheter from the overhead microscope. Note that small clots are visible near the tip of the catheter.

In this study, the Automated, *In Vitro* Model for Hydrocephalus Research (AIMS) was utilized to investigate ventricular catheter failure, modified to include blood in solution (Figures 1A,B). The development of AIMS and bioreactor chambers was reported in detail in previous studies (Faryami et al., 2022; Menkara et al., 2022; Faryami et al., 2024). Briefly, AIMS was composed of three main components for this study: (1) Polyethylene terephthalate glycol (PETG) transparent chambers, (2) Python program and control unit (3) Custom-built positive displacement pumps. The PETG chambers were fabricated utilizing a vacuum forming machine (A3, Vacucu 3D, China) and a 3D printed resin mold (Photon 4K MSLA, Anycubic Technology CO., Limited, Hong Kong). Ventricular catheters and EVDs were cut 40 mm away from the tip and were placed in the chambers (Figures 1C,D).

The Control Unit consisted of an Arduino-based controller board and a custom-built, proprietary Python program with a custom user interface. The Python program transformed the user input (Pulsation Rate, Bulk Flow Rate, Systole Time) into real-time commands that controlled the motion of the pumps (Figure 1A). The pumps interfaced directly with the fluidic components, including check valves and silicone tubing, to instigate flow through PETG chambers.

The pumps, check valves (Duckbill Check Valve, Qosina, USA), bioreactor chamber, and flow sensor (SLF3s-1300F Series, Sensirion, Switzerland) were all placed in series in a circuit. A pressure manometer (Extech HD700 Differential Pressure Manometer - 2PSI, Teledyne FLIR LLC, USA) was placed at the inlet of each PETG chamber. Sensirion USB Sensor Viewer (Sensirion, Switzerland) and HD700 Data Acquisition software (Teledyne FLIR LLC, USA) were utilized to monitor flow and pressure in real time (Figure 1A).

In tandem with quantitative measurements, a Trinocular stereo zoom brightfield microscope (SM-4TZ-144A, AmScope, USA) and the high-throughput imaging setup of AIMS with digital microscopes (Plugable Technologies, USA) were utilized to record clot formation in real-time. Frames from the recording of the experiments were exported for further analysis (Figure 1E). The speed of the video recordings was increased by 250 times to visualize clot formation (Vegas Pro. 17, Magix, Germany). The Trinocular stereo zoom microscope was also utilized to capture the presence and the distribution of clots on the catheters post-hoc. The lumen of the catheters was exposed by cutting along the lateral holes. All the programs ran on a Hewlett-Packard desktop computer running Windows 10 Pro operating system.

### Experimental protocol

To investigate our hypotheses, shunt failure was investigated in nine groups of catheters (n = 4 per group) that were divided into the following categories: 1) Control, 2) Blood Concentration, 3) Bulk Flow Rate, and 4) Catheter Type (Table 1). Twenty-four antibiotic-impregnated ventricular catheters, four barium-impregnated ventricular catheters, and eight antibiotic-impregnated EVDs were utilized in this investigation. The make and model of the ventricular catheters remained consistent in groups marked as “Antibiotic” under the Catheter Type column. The make and the architecture of antibiotic EVDs were also consistent, except that in the EVD-0.5% group, the antibiotic EVDs were opaque, and in the EVD-5% group, the antibiotic EVDs were of the clear variant.

**TABLE 1:** The summary of the groups included in the study. The list of groups and variables investigated.

Conditions	Sample size (N)	Group name	Concentration blood (%)	Flow (mL/min)	Catheter type
Control	4	Saline	0	0.3	Antibiotic
	4	Heparinized Blood	0.5	0.3	Antibiotic
Blood Concentration	4	Low Concentration	0.05	0.3	Antibiotic
	4	High Concentration	0.5	0.3	Antibiotic
Bulk Flow Rate	4	High Flow Rate	0.5	0.7	Antibiotic
	4	High Flow Rate- Low Blood Concentration	0.05	0.7	Antibiotic
Catheter Type	4	Barium-Impregnated	0.5	0.3	Barium
	4	EVD-0.5%	0.5	0.3	EVD-antibiotic
	4	EVD-5%	5	0.3	EVD-antibiotic-Clear

Saline (0.9% sodium chloride) was utilized to dilute heparinized whole porcine blood to prepare blood-saline solutions at two concentrations of 0.05 and 0.5 Volume percent (v/v%). In the control category, four antibiotic ventricular catheters were exposed to saline only (Saline group) conducted at 0.3 $\frac{mL}{\min}$ , and four antibiotic catheters were exposed to 0.5% heparinized blood (Heparinized Blood group).

#### Blood concentration

In this study, a blood-laden solution was used to identify the effects of continual exposure to blood. A 0.05% and 0.5% blood-saline concentrations were used, and derived from clinical reports of red blood cell (RBC) count in CSF of IVH patients (Fam et al., 2017; Javinsky, 2012). Catheters that were exposed to 0.5% blood-saline solution delivered at 0.3 $\frac{mL}{\min}$ bulk flow rate were classified as the High Concentration group and catheters that were exposed to 0.05% concentration at 0.3 $\frac{mL}{\min}$ were categorized as the Low Concentration Group. One liter of solution was prepared for each sample consisting of equal parts of Solution A and Solution B. Solution A contained saline and heparinized whole porcine blood, and Solution B contained saline, thrombin at 11 $\frac{NIH\;units}{mL\;Blood}$ (Sigma-Aldrich, USA), and protamine sulfate $\frac{10\;mg}{mL\;Blood}$ (Sigma-Aldrich, USA). Whole Blood samples were mixed with heparin (10 $\frac{USP\;units}{mL}$ ) before shipment. The inlet of each shunt-containing chamber interfaced with two pump channels to simultaneously deliver two separate solutions (Solution A and Solution B) (Figure 1A).

Solution A and Solution B were contained in separate reservoirs for the duration of the experiment. The pumps automatically pulled from the reservoirs, and the output of the channels merged immediately before the chamber inlet. Note that in the saline control group, both reservoirs were filled with saline. Similarly, in the blood control group, thrombin or protamine sulfate was not added to Solution B, and only heparinized blood came in contact with the catheters. Whole porcine blood was sourced from licensed suppliers in compliance with established ethical standards and regulatory guidelines. All data collection was performed within 1 week of heparinized porcine blood delivery.

#### Bulk flow rate

The impact of bulk flow rate on ventricular catheter obstruction was also investigated in this study (Table 1). The custom Python user interface was used to set the combined bulk output of the two synchronized concurrent channels to 0.3 $\frac{mL}{\min}$ and 0.7 $\frac{mL}{\min}$ (Liu et al., 2022; Battal et al., 2011). All samples were exposed to pulsatile flow at 70 $\frac{Beats}{\min}$ pulsation rate. The physiologic bulk flow rate of 0.3 $\frac{mL}{\min}$ was conducted through all samples except the samples in high flow rate groups. The bulk flow rate was set to 0.7 $\frac{mL}{\min}$ for samples in the High Flow Rate group and High Flow Rate- Low Blood Concentration group. The delivered bulk flow was confirmed via flow sensor and volumetric analysis using a Mettler Toledo AT261 DeltaRange Analytical Balance (Mettler-Toledo, LLC, USA).

#### Catheter type

The four barium-impregnated ventricular catheters in the Barium-impregnated group and the four EVDs in the EVD-0.5% group were exposed to 0.5% blood-solution conducted at 0.3 $\frac{mL}{\min}$ bulk flow rate, the same as the antibiotic-impregnated ventricular catheters in the High Concentration group. The four EVDs in the EVD-5% group were exposed to the most concentrated blood-saline solution at 5% (v/v%).

The AIMS’s automated priming feature facilitated constant fluid contact throughout the study. Pressure and flow data acquisition was initiated simultaneously upon completion of the priming process. The brightfield recording was also started at this time point. AIMS was programmed to terminate the experiment automatically after 24 h. The shunt failure criteria were set at 30 ${cmH}_{2}O$ . The experiment was manually terminated if pressure surpassed 30 ${cmH}_{2}O$ differential pressure at any point within 24 h. The degree of obstruction of the catheters was quantified using four measurable outcomes: (1) The mean of maximum pressure reached during the experiment (2) The average survival time (3) the number of catheters in the group that failed during the experiment (4) The rate at which the maximum pressure was reached (Equation 1). Pressurization rate was utilized in all statistical analysis.

$$Pressurization\;Rate=\frac{Maximum\;Recorded\;Pressure-Baseline\;Pressure\;\left({cmH}_{2}O\right)}{Time\;\left(Hrs\right)}$$ (1)

### Statistical analysis

GraphPad Prism v10.0 (GraphPad Software, USA) was utilized for data curation and visualization. Statistical analyses were performed using SPSS Statistics Suite (IBM Corporation, USA). The Kolmogorov–Smirnov test and the Shapiro–Wilk test were performed to investigate the normality of data. The Levene Test of Homogeneity of Variances was performed to evaluate the homoscedasticity of the datasets. For all tests, a confidence interval was set at 0.95 (α = 0.05). Non-parametric data were analyzed using Mann-Whitney U and Kruskal–Wallis with pairwise multiple comparisons. Significance values were adjusted by the Bonferroni correction for multiple tests (Adj. Sig.).

## Results

**FIGURE 2:**
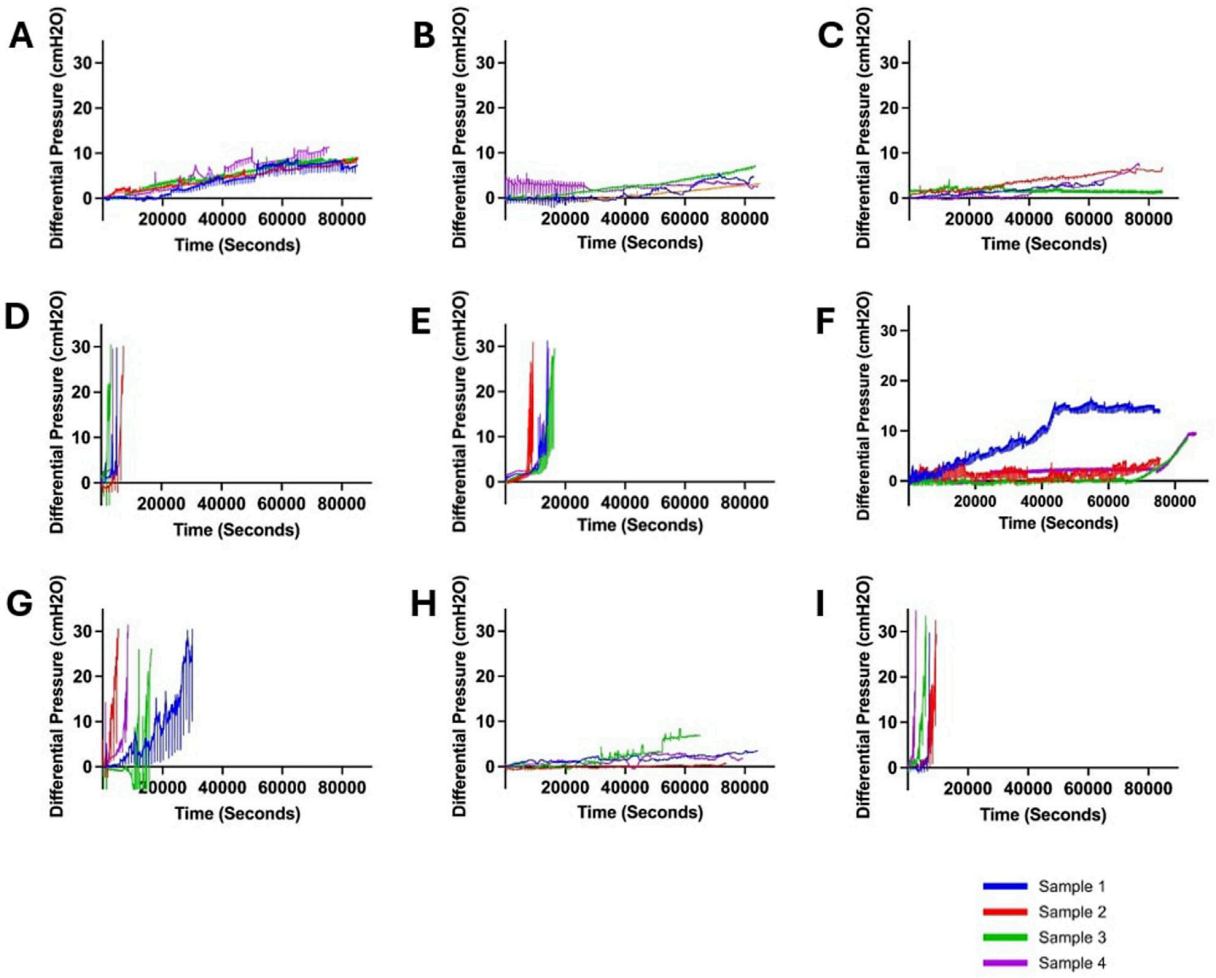
Visualization of changes in differential pressure (cmH_2_O) for all ventricular catheter and EVD groups (N = 4 per group). **(A)** Saline Group. Antibiotic ventricular catheters exposed to saline solution at 0.3 mL/min bulk flow rate did not reach the 30 cmH_2_O threshold within 24 h. **(B)** Heparinized Blood. Antibiotic ventricular catheters exposed to 0.5% concentration of heparinized blood at 0.3 mL/min bulk flow rate did not reach the 30 cmH_2_O threshold within 24 h. **(C)** Low Concentration (0.05%) Blood Solution. Antibiotic ventricular catheters exposed to a 0.05% concentration of blood and thrombin solution at 0.3 mL/min bulk flow rate did not reach the 30 cmH_2_O threshold within 24 h. **(D)** High Concentration (0.5%) Blood Solution. All the antibiotic ventricular catheters exposed to a 0.5% concentration of blood and thrombin solution at 0.3 mL/min reached the 30 cmH_2_O threshold within 6,372 ± 1152 s. **(E)** High Flow Rate (0.7 mL/min). All the antibiotic ventricular catheters exposed to a 0.5% concentration of blood and thrombin solution at 0.7 mL/min bulk flow rate reached the 30 cmH_2_O threshold within 109,620 ± 1,908 s. **(F)** High Flow Rate- Low Blood Concentration. Antibiotic ventricular catheters exposed to a 0.05% concentration of blood and thrombin solution at 0.7 mL/min bulk flow rate did not reach the 30 cmH_2_O threshold within 24 h. **(G)** Barium-Impregnated. All barium-impregnated ventricular catheters exposed to a 0.5% concentration of blood and thrombin solution at 0.3 mL/min reached the 30 cmH_2_O threshold within 16,380 ± 8,532 s. **(H)** EVD-0.5%. The antibiotic EVDs exposed to a 0.5% concentration of blood and thrombin solution at 0.3 mL/min did not reach the 30 cmH_2_O threshold within 24 h. **(I)** EVD-5%. All the antibiotic EVDs exposed to a 5% concentration of blood and thrombin solution at 0.3 mL/min reached the 30 cmH_2_O threshold within 6,588 ± 2,916 s.

Overall, 36 samples were investigated in this study. More than 500 h of quantitative and qualitative data were collected. No leaks or sudden ruptures were observed. Notable differences in peak pressure, survival time, and pressurization rate were observed across all ventricular catheter and EVD groups (Figure 2). All the samples in the Saline (Figure 2A), Heparinized Blood (Figure 2B), Low Concentration (0.05%) Blood Solution (Figure 2C), High Flow Rate- Low Blood Concentration (Figure 2F), and EVD-0.5% (Figure 2H) groups completed the 24 Hr exposure to pulsatile flow because the 30 ${cmH}_{2}O$ threshold was not reached.

In contrast, all the samples in the High Concentration (0.5%) Blood Solution group (Figure 2D), High Flow Rate (0.7 $\frac{mL}{\min}$ ) group (Figure 2F), Barium-Impregnated group (Figure 2G), and EVD-5% (Figure 2I) were manually terminated when the 30 ${cmH}_{2}O$ threshold was reached.

### Ventricular catheter failure and blood concentration

**FIGURE 3:**
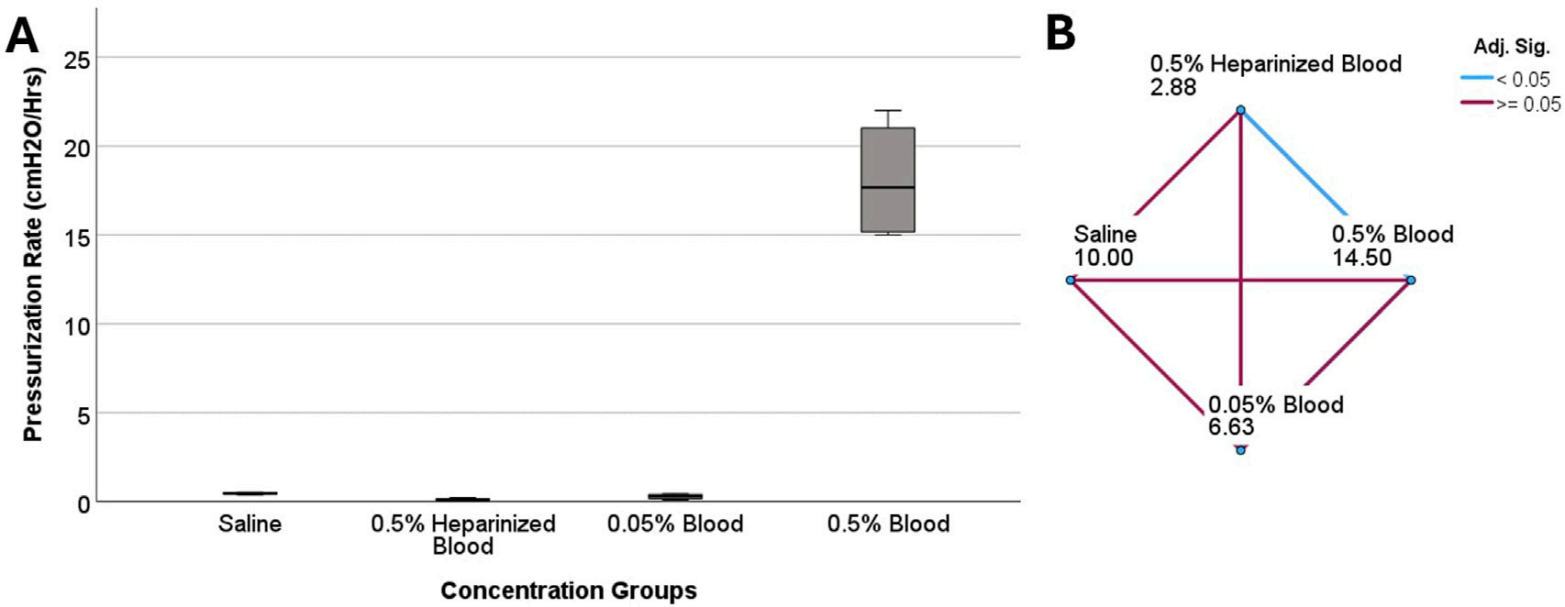
The investigation of differences in pressurization rate across blood concentration groups in ventricular catheters. **(A)** The boxplot illustrates the difference in pressurization rate across the groups. A statistically significant difference in pressurization rate was observed (*P = 0.005*) **(B)** Pairwise comparison of concentration groups. Note that each node shows the sample average rank of the group. A statistically significant difference was observed between 0.5% heparinized blood and 0.5% blood groups (Adj. Sig. = 0.003).

All control samples completed the 24-hour experiments (Table 2). The mean of maximum pressure was 9.72 ± 0.74 ${cmH}_{2}O$ and 2.35 ± 1.81 ${cmH}_{2}O$ for saline and heparinized blood, respectively. A slight increase in peak pressure was observed in the 0.05% Blood group at 4.77 ± 2.7 ${cmH}_{2}O$ . However, the threshold for shunt failure was not reached, and all four samples completed the 24-hour experiment. In contrast, all the samples in the 0.5% blood group reached the 30 ${cmH}_{2}O$ threshold within 1.77 ± 0.32 h. There was a statistically significant difference in the pressurization rate across the blood concentration groups (*P = 0.005*) (Figure 3). In the pairwise comparison of concentration groups, a statistically significant difference was observed between 0.5% heparinized blood and 0.5% blood groups (Adj. Sig. = 0.003) (Figure 3B). Similarly, a statistically significant difference was observed between EVDs exposed to 0.5% blood concentration and 5% blood concentration (Supplementary Figure S1). While the EVDs exposed to 0.5% blood-saline solution completed the 24-h experiment with a peak pressure of 2.05 ± 0.70 ${cmH}_{2}O$ , a peak pressure of 30.72 ± 0.52 ${cmH}_{2}O$ was recorded for EVDs exposed to the 5% blood concentration. The average survival time for EVDs in the 5% blood concentration group was 1.83 ± 0.81 Hrs.

**TABLE 2:** The summary of the results. The list of groups and variables investigated, the number of failed catheters, mean of maximum pressure achieved, average survival time and mean pressurization rate.

Group name	Number of failed catheters	Mean of maximum pressure ±SD (cmH_2_O)	Average survival Time± SD (Hrs.)	Mean of pressurization Rate± SD (cmH_2_O/Hrs.)
Saline	0	9.72 ± 0.74	24 ± 0	0.47 ± 0.06
Heparinized Blood	0	2.35 ± 1.81	24 ± 0	0.1 ± 0.08
Low Concentration (0.05%) Blood	0	4.77 ± 2.7	24 ± 0	0.29 ± 0.16
High Concentration (0.5%) Blood	4	31.3 ± 1.54	1.77 ± 0.32	18.08 ± 3.47
High Flow Rate (0.7 mL/min)	4	30.45 ± 0.53	4.2 ± 1.46	8.04 ± 3.13
High Flow Rate- Low Blood Concentration	0	9.57 ± 2.71	24 ± 0	0.45 ± 0.13
Barium-Impregnated	4	31.5 ± 1.93	4.55 ± 2.37	8.49 ± 4.08
EVD-0.5%	0	2.05 ± 0.70	24 ± 0	0.1 ± 0.03
EVD-5%	4	30.72 ± 0.52	1.83 ± 0.81	21.65 ± 14.55

### Ventricular catheter failure and bulk flow rate

**FIGURE 4:**
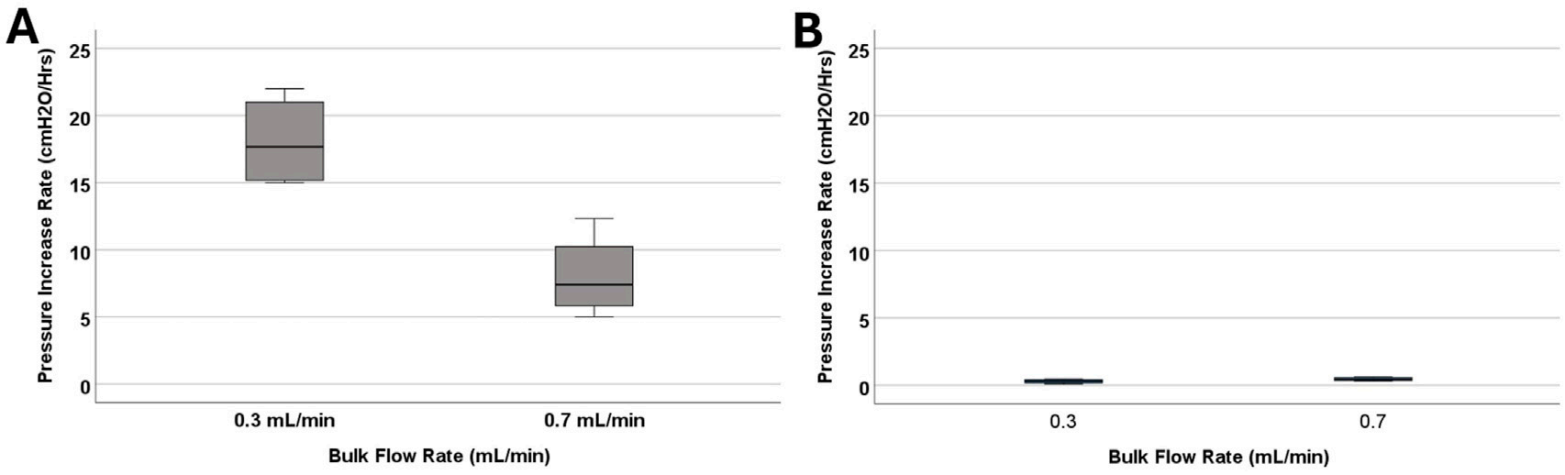
The investigation of differences in pressurization rate across bulk flow rate groups. **(A)** The boxplot illustrates the difference in pressurization rate across the 0.1 mL/min, 0.3 mL/min, and 0.7 mL/min groups. Note that blood concentration remained constant across the groups at 0.5%. A statistically significant difference in pressurization rate was observed (*P = 0.029*). **(B)** The boxplot illustrates the difference in pressurization rate across the 0.3 mL/min, and 0.7 mL/min groups. Note that blood concentration remained constant across the groups at 0.05%. No statistically significant difference in pressurization rate was observed (*P = 0.2*).

Although all four samples that were exposed to 0.7 $\frac{mL}{\min}$ reached the threshold of 30 ${cmH}_{2}O$ similar to those exposed to 0.3 $\frac{mL}{\min}$ , the average survival time for the High Flow Rate (0.7 $\frac{mL}{\min}$ ) group was 4.2 ± 1.46 h; comparatively higher than samples exposed to 0.3 $\frac{mL}{\min}$ with a survival time of 1.77 ± 0.32 h. There was a statistically significant difference in pressurization rate between the groups (*P = 0.029*) (Figure 4A).

The impact of bulk flow rate on pressurization rate was also investigated in low blood concentration groups (Figure 4B). All the catheters that were exposed to 0.05% concentration blood solution completed the 24-h experiment. The samples that were exposed to 0.7 $\frac{mL}{\min}$ reached a maximum pressure of 9.57 ± 2.71 ${cmH}_{2}O$ on average, slightly higher than the samples exposed to 0.3 $\frac{mL}{\min}$ with 4.77 ± 2.7 ${cmH}_{2}O$ . However, no statistically significant difference was observed between the groups that were exposed to 0.05% blood solution at 0.3 $\frac{mL}{\min}$ and 0.7 $\frac{mL}{\min}$ (P = 0.2) within the specified 24-hour period.

### Ventricular catheter type

**FIGURE 5:**
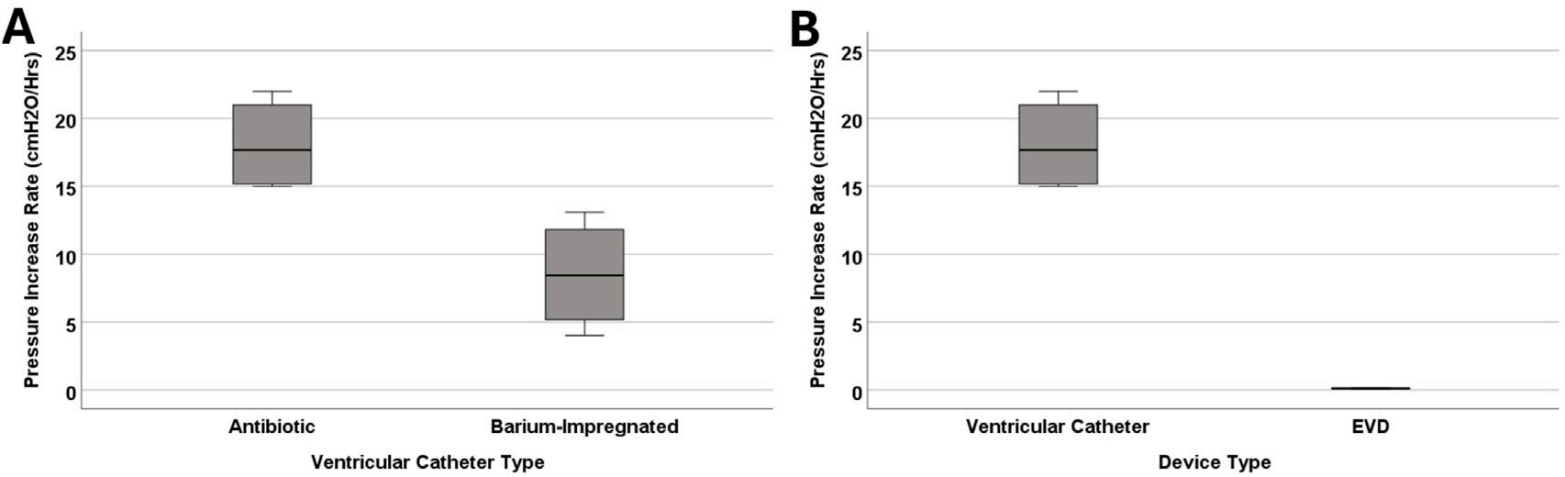
The investigation of differences in pressurization rate in catheter types. **(A)** The boxplot illustrates the difference in pressurization rate between antibiotic and barium-impregnated catheters. A statistically significant difference was observed between antibiotic and barium-impregnated ventricular catheters (*P = 0.029*). **(B)** The boxplot illustrates the difference in pressurization rate between antibiotic ventricular catheters and EVDs. A statistically significant difference was observed between ventricular catheters and EVDs (*P = 0.029*). Note that blood concentration remained constant across the groups at 0.5% for all the groups represented in this figure.

Similar to antibiotic-impregnated catheters, all the barium-impregnated ventricular catheters reached the failure threshold when exposed to 0.5% blood concentration solution at 0.3 $\frac{mL}{\min}$ bulk flow rate. However, the average survival time of barium-impregnated catheters was more than twice as long as antibiotic-impregnated catheters at 4.55 ± 2.37 h and 1.77 ± 0.32 h, respectively. There was a statistically significant difference in the pressurization rate between barium-impregnated catheters and antibiotic-impregnated catheters (*P = 0.029*) (Figure 5A).

A more drastic difference was observed between the antibiotic-impregnated ventricular catheters and antibiotic-impregnated EVDs (Figure 5B). All four EVDs completed the 24-hour experiment and did not reach the threshold. The mean of peak pressure for EVDs was 2.05 ± 0.7 ${cmH}_{2}O.$ In contrast, all four antibiotic-impregnated ventricular catheters that were exposed to 0.5% blood solution reached the 30 ${cmH}_{2}O$ threshold in 1.77 ± 0.32 Hrs on average. There was a statistically significant difference in pressurization rate between the EVDs and ventricular catheters (*P = 0.029*).

**FIGURE 6:**
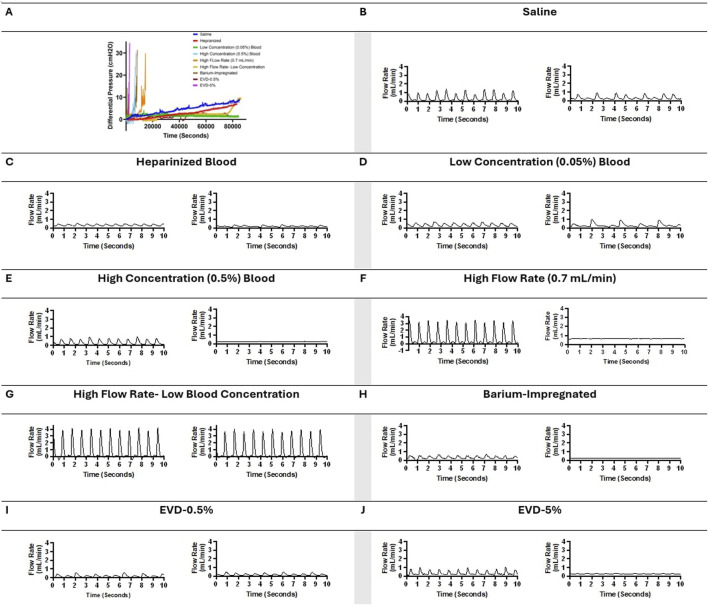
Visual representation of changes in pressure and flow over time across all the groups in representative samples. **(A)** Change in differential pressure over time across samples from all the groups. **(B)** A ten-second segment of flow measurement in a sample in the saline group shortly after the initiation of flow and a ten-second segment of flow measurement in the same sample in the saline group shortly before termination of the experiment after 24 h of exposure to pulsatile flow. **(C)** A ten-second segment of flow measurement in a sample in the heparinized blood group shortly after the initiation of flow and a ten-second segment of flow measurement in the same sample in the heparinized blood group shortly before termination of the experiment after 24 h of exposure to pulsatile flow. **(D)** A ten-second segment of flow measurement in a sample in the Low Concentration (0.05%) Blood group shortly after the initiation of flow and a ten-second segment of flow measurement in the same sample in the Low Concentration (0.05%) group shortly before termination of the experiment after 24 h of exposure to pulsatile flow. **(E)** A ten-second segment of flow measurement in a sample in the High Concentration (0.5%) Blood group shortly after the initiation of flow and a ten-second segment of flow measurement in the same sample in the High Concentration (0.5%) group shortly before termination of the experiment because the 30 cmH_2_O threshold was reached. **(F)** A ten-second segment of flow measurement in a sample in the High Flow Rate (0.7 mL/min) Blood group shortly after the initiation of flow and a ten-second segment of flow measurement in the same sample in the High Flow Rate (0.7 mL/min) group shortly before termination of the experiment because the 30 cmH_2_O threshold was reached. **(G)** A ten-second segment of flow measurement in a sample in the High Flow Rate- Low Blood Concentration group shortly after the initiation of flow and a ten-second segment of flow measurement in the same sample in the High Flow Rate- Low Blood Concentration shortly before termination of the experiment after 24 h of exposure to pulsatile flow. **(H)** A ten-second segment of flow measurement in a sample in the Barium-Impregnated group shortly after the initiation of flow and a ten-second segment of flow measurement in the same sample in the Barium-Impregnated group shortly before termination of the experiment because the 30 cmH_2_O threshold was reached. **(I)** A ten-second segment of flow measurement in a sample in the EVD-0.5% group shortly after the initiation of flow and a ten-second segment of flow measurement in the same sample in the EVD-0.5% group shortly before termination of the experiment after 24 h of exposure to pulsatile flow. **(J)** A ten-second segment of flow measurement in a sample in the EVD-0.5% group shortly after the initiation of flow and a ten-second segment of flow measurement in the same sample in the EVD-5% group shortly before termination of the experiment because the 30 cmH_2_O threshold was reached.

The waveforms in Figures 6B–J illustrated the impact of obstruction formation and device patency on pulsatile flow through the devices investigated in this study. Consistent with Figure 2, a notable difference was observed between the groups that reached the 30 ${cmH}_{2}O$ and the samples that did not such that no major changes in the flow waveform were observed in the groups that completed the 24-h experiment without reaching the 30 ${cmH}_{2}O$ threshold (Figures 6B–D,G,I). However, in the groups that reached the 30 ${cmH}_{2}O$ , ten-second segments recorded shortly before reaching the 30 ${cmH}_{2}O$ demonstrated a considerable decrease in pulsatility and amplitude compared to the waveform observed shortly after the initiation of flow. (Figures 6E,F,H,J).

### Qualitative analysis

**FIGURE 7:**
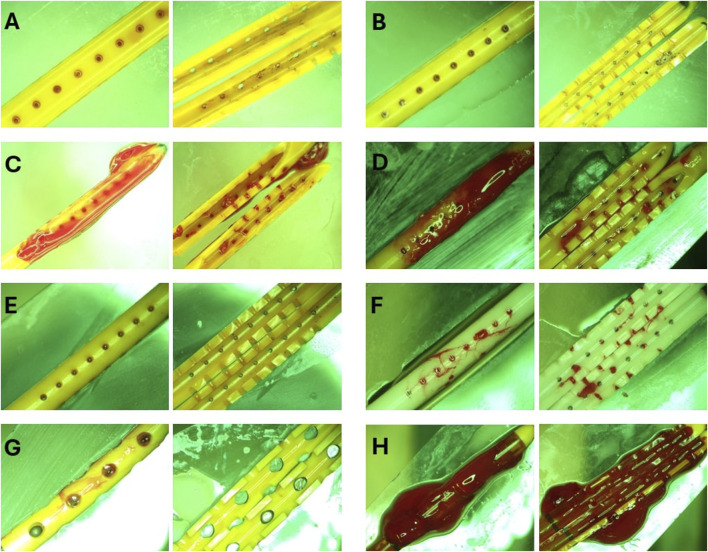
Representative End point view of catheters external surfaces and lumen. **(A)** Heparinized Blood. No blood clots were visible inside or outside the catheter. **(B)** Low Concentration (0.05%) Blood Solution. Small blood clots were visible in the cross-section of the lateral holes. Small clots were also observed on the outer surface of the catheters similar to the blood clot visible in Figure 1E. **(C)** High Concentration (0.5%) Blood Solution. Extensive clot formation was observed inside and outside of the catheter with blood clots expanding through the lateral holes. **(D)** High Flow Rate (0.7 mL/min). Extensive clot formation was observed inside and outside the catheter. **(E)** High Flow Rate- Low Blood Concentration. No blood clots were visible inside or outside the catheter **(F)** Barium-Impregnated. Relatively large blood clots were observed inside the lumen of the catheter. Clots were also observed expanding through the lateral holes of the catheter. **(G)** EVD-0.5%. Loosely associated blood clots were observed on the surface of the EVDs. **(H)** EVD-5%. The EVD was engulfed in a large continuous blood clot expanding through the lumen and outer surface of the EVD.

The post-hoc visual evaluation of ventricular catheter and EVD patency was consistent with quantitative measurements such that catheters and EVDs from groups that reached the threshold demonstrated visible clots on the external surfaces and the lumen of the devices (Figure 7). Clots were not observed in ventricular catheters from Heparinized blood (Figure 7A) and High-flow rate-Low Concentration (Figure 7E). Minimal clotting was observed in the lateral holes of ventricular catheters in Low Concentration (0.05%) Blood Solution (Figure 7B). Additionally, loosely associated blood clots were observed on the surface of the EVDs in EVD-0.5% group (Figure 7G). In contrast, extensive clot formation was in samples of the High Concentration (0.5%) Blood Solution group with clots extending inside and outside of the catheter, with blood clots expanding through the lateral holes (Figure 7C). Extensive clot formation was also observed in samples of High Flow Rate (0.7 $\frac{mL}{\min}$ ) (Fig. D). Similarly, intraluminal clots were observed in barium-impregnated ventricular catheters. Blood clots were also observed in the cross-section of lateral holes. However, the clots on the external surfaces of the catheters were less extensive compared to antibiotic-impregnated ventricular catheters (Figure 7F). Finally, the most extensive thrombosis was observed in EVDs exposed to 5% blood solution. The EVDs were engulfed in large, continuous blood clots expanding through the lumen and outer surface of the EVDs (Figure 7H).

**FIGURE 8:**
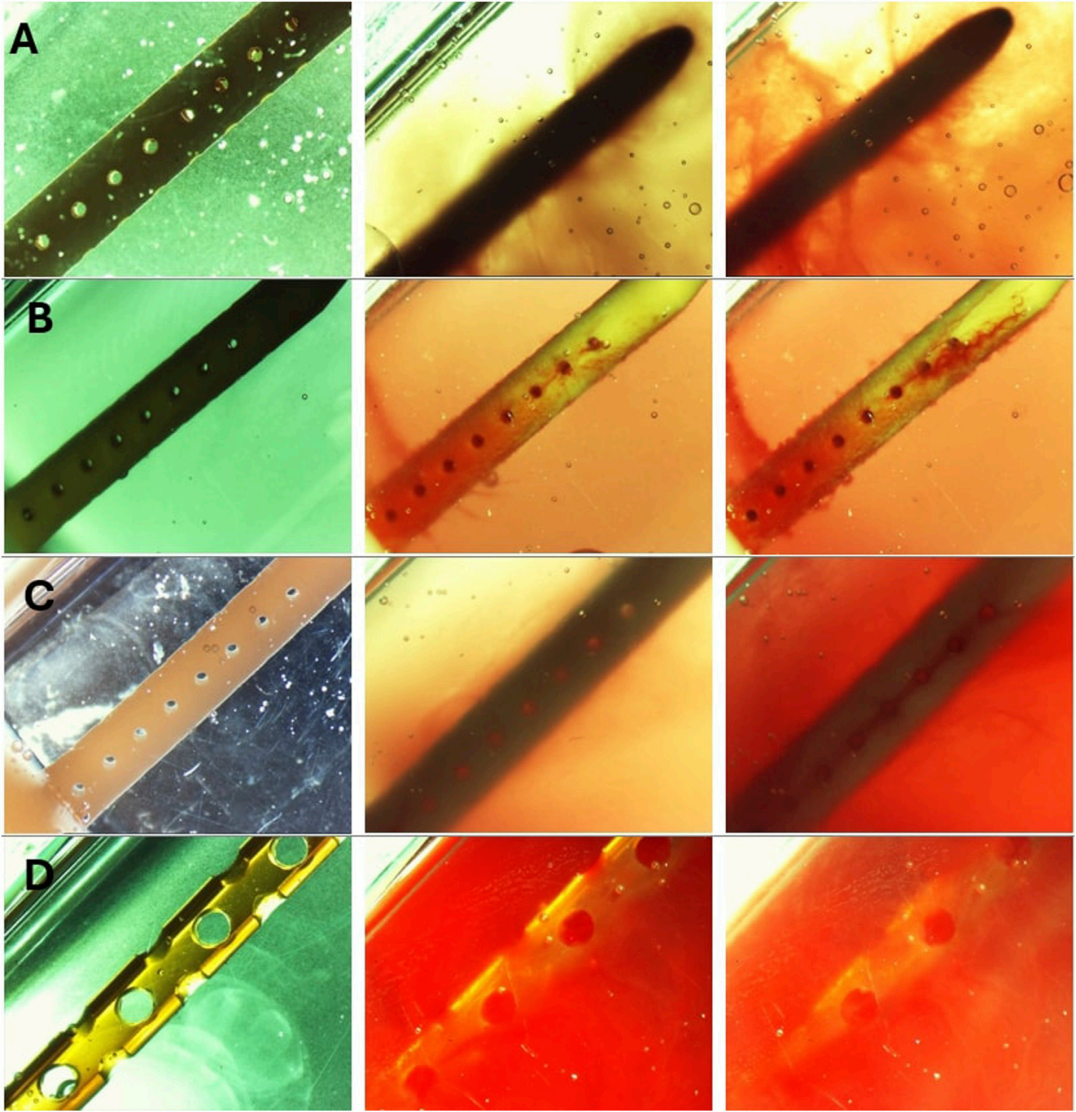
**T**imelapse view of ventricular catheters and EVDs in samples that reached the 30 cmH_2_O threshold. **(A)** High Concentration (0.5%) Blood Solution. The antibiotic ventricular catheters exposed to a 0.5% concentration of blood and thrombin solution at 0.3 mL/min showed extensive web-like clot formation throughout the catheter. **(B)** High Flow Rate (0.7 mL/min). Extensive clot formation was observed in the vicinity of lateral holes furthest from the catheter in the antibiotic ventricular catheter exposed to a 0.5% concentration of blood and thrombin solution at 0.7 mL/min bulk flow rate. Note that fine strands were observed expanding between lateral holes. **(C)** Barium-Impregnated. A fine web-like blood clot was observed around the Barium-impregnated ventricular catheter that was exposed to a 0.5% concentration of blood and thrombin solution at 0.3 mL/min. **(D)** EVD-5%. The EVD exposed to a 5% at 0.3 mL/min bulk flow rate was rapidly engulfed in a continuous blood clot. The 5% blood solution significantly reduced the visibility of the EVD in the PETG chamber.

Post-hoc evaluation of ventricular catheters and EVDs offered qualitative insights into the differences between patent and obstructed devices. Time-lapse videos and pictures, on the other hand, provided insights into the mechanisms of obstruction formation (S. Vid. 1-4). Note that the visibility of the catheters and EVDs was highly influenced by the concentration of the blood solution, such that catheters exposed to higher blood concentration (Figure 8D) were less visible than samples that were exposed to a lower concentration of blood (Figures 8A–C). Additionally, the visibility of the catheters was also significantly impacted by bulk flow rate, such that catheters exposed to a higher bulk flow rate (Figure 8B) were more visible than catheters at a lower bulk flow rate (Figures 8A,C).

The development of a fibrin sheath was noted as one of the first steps in catheter obstruction in the samples in the High Concentration (0.5%) Blood Solution group and the Barium-Impregnated group (Figures 8A,C) (Supplementary Video S1, S3). In Supplementary Video S1, the development of the fibrin sheath appeared to be localized in the vicinity of specific lateral holes and appeared to switch between different lateral holes throughout the video. Thrombosis was observed near the lateral holes of the catheters, forming a fibrotic bridge across the lateral holes of the catheters (Figures 8B,C) (Supplementary Figure S2). Directionality was observed in the fibrotic bridge expansion such that the clot from the third hole from the tip expanded and connected with the second lateral hole from the catheter tip as the blood clots form across the holes (Supplementary Figure S2).

Finally, clot formation was observed near the tip of the catheters (Fig. A, B). While in the sample from the High Concentration group (Supplementary Video S1), precipitous and extensive sheath formation was observed throughout the catheter, the sheath formation in the barium-impregnated sample (Supplementary Video S3) appeared near the lateral holes furthest from the tip of the catheter and gradually expanded to the middle section of the catheter.

**FIGURE 9:**
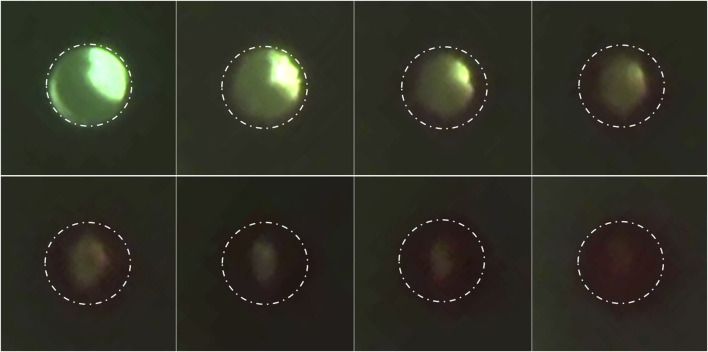
Timelapse of lateral hole obstruction in a barium-impregnated ventricular catheter. Zoomed in frames from Supplementary Video S3 illustrating the process of obstruction formation on the lateral holes of the barium-impregnated ventricular catheter. The white dotted line shows the rough outline of the lateral hole. The round unobstructed lateral hole gradually became obstructed with blood in an outside-in process. The obstruction process began with the coating of the walls of the lateral hole. Building on the initial layer, the clot formation expanded inwards towards the center of the hole and gradually reduced the effective diameter of the lateral hole until complete obstruction was achieved. Note that the brightness was increased in post-processing to improve the visibility of the clot formation.

The obstruction formation in the lateral holes of the barium-impregnated catheter also appeared to follow a similar trend, with the lateral holes furthest from the tip showing signs of obstruction formation and gradually expanding up towards the tip. While the obstruction of the lateral holes was visible in Supplementary Videos S1, S2, it was most visible in Supplementary Video S3. Zoomed-in frames from Supplementary Video S3 demonstrated the process of obstruction formation on the lateral holes of the barium-impregnated ventricular catheter (Figure 9). The round, unobstructed lateral hole gradually became obstructed with blood in an outside-in process. The obstruction process began with the coating of the walls of the lateral hole. Building on the initial layer, the clot formation expanded inwards towards the center of the hole and gradually reduced the effective diameter of the lateral hole until complete obstruction was achieved. Note that this process did not occur at the same rate for all the holes in this row of lateral holes.

## Discussion

Our previous investigations demonstrated the capability of AIMS to flow CSF-like solution through a tubing, a chamber, and catheters with up to 50 synchronized independent pump channels (Faryami et al., 2024). In this study, each chamber was interfaced with two independent channels, effectively reducing the high throughput capability of the setup by 50 percent (Figure 1). Despite the reduction in high-throughput capabilities, the controlled introduction of hematoma setup with two synchronized concurrent channels addressed the variations associated with manual administration of boluses such as bolus volume, mixing rate, and delivery rate of bolus injections (Younger et al., 2023). Additionally, during our preliminary experiments, we ran Solution A and Solution B through empty PETG chambers for 24 h. The chamber and tubing did not elicit clot formation by themselves over this period.

One of the main difficulties in investigating shunt failure is the lack of a well-documented definition of shunt obstruction. In this study, 30 ${cmH}_{2}O$ was set as the criterion for shunt failure based on clinical reports of severe and life-threatening patient symptoms at that elevated intracranial pressure (ICP) (Bø and Lundqvist, 2020; Kareemi et al., 2023; Zacchetti et al., 2015). The average ICP is reported between 6-25 ${cmH}_{2}O$ despite rare cases where individuals with ICP above 30 ${cmH}_{2}O$ have no apparent symptoms (Lee and Lueck, 2014). Variations in the physiology and pathophysiology of different patient groups are another complication associated with investigating shunt obstruction. More studies are required to investigate shunt failure in specific PHH patient groups, particularly patients with co-morbidities.

The results presented in Figures 2, 3 also highlight the importance of thrombosis on the patency of the samples in this investigation. While the samples in the heparinized blood and High Concentration (0.5%) Blood Solution were exposed to the same 0.5% blood concentration solution, the peak pressure in the heparinized blood group was only 2.35 ± 1.81 ${cmH}_{2}O$ while all the samples that were exposed to thrombin, protamine sulfate, and the same concentration of blood solution reached the 30 ${cmH}_{2}O$ threshold within 1.77 ± 0.32 Hrs. These results might suggest that drugs that specifically target thrombosis may be great targets for shunt modification with bioactive agents. However, anticoagulants also have a strong association with intracranial hemorrhages (Fargen et al., 2016) such that a previous retrospective clinical investigation identified aspirin intake as a patient risk factor for EVD-related hemorrhage (Woo et al., 2019). Another clinical study reported therapeutic anticoagulation as a predictor of EVD occlusion (Fargen et al., 2016). Interestingly, several previous studies investigated the impact of blood thinners in central venous catheters, which could provide invaluable insights into improving the longevity of ventricular catheters (Haire and Herbst, 2000; Klerk et al., 2003; Rezazadeh et al., 2022; Wall et al., 2016). These results suggest that future studies are warranted to investigate the long-term impact of anticoagulants and antiplatelets on ventricular catheter survival.

This study demonstrates that the concentration of blood in saline solutions was positively correlated with the pressurization rate in ventricular catheters and EVDs. Shunts and EVDs that were exposed to higher concentrations of blood in saline consistently reached the threshold more than samples exposed to low concentrations of blood in saline solution (Figure 3) (Supplementary Figure S1). These results support our hypothesis that ventricular catheters fail more rapidly as the concentration of blood in saline solution increases. Our results also indicated that CSF bulk flow rate had an impact on shunt obstruction. Contrary to our initial hypothesis, a decrease in the pressurization rate was observed when comparing 0.7 $\frac{mL}{\min}$ bulk flow rate groups to 0.3 $\frac{mL}{\min}$ flow rate (Figure 4). The longer survival time of the samples exposed to pulsatile flow at 0.7 $\frac{mL}{\min}$ (4.2 ± 1.46 Hrs) compared to 1.77 ± 0.32 Hrs in samples exposed to pulsatile flow at 0.3 $\frac{mL}{\min}$ provided preliminary evidence of the importance of bulk flow rate on thrombi formation in ventricular catheter failure. Blood flow is widely considered one of the important variables that contribute to thrombosis. While the investigation of flow and shear-mediated thrombosis is an active area of research, our results agree with several previous studies that reported the inhibitory effect of high shear on clot formation in benchtop and *in vivo* experiments (Shibeko et al., 2010; Weiss et al., 1986; Shibeko et al., 2009).

**FIGURE 10:**
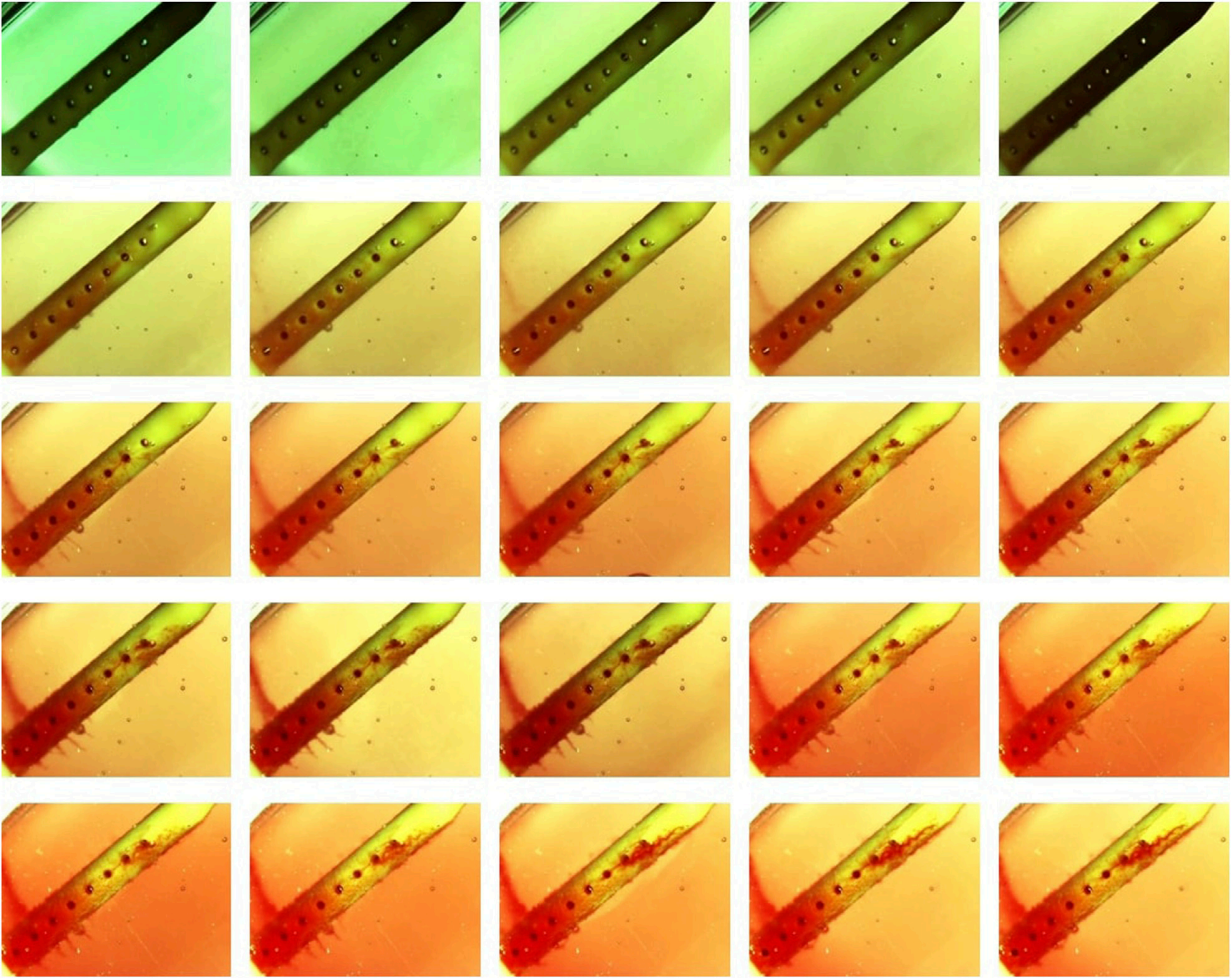
Detailed timelapse of clot formation. Antibiotic ventricular catheter was exposed to a 0.5% concentration of blood and thrombin solution at 0.7 mL/min bulk flow rate. Clot formation was more apparent near the lateral holes furthest from the catheter tip.

**FIGURE 11:**
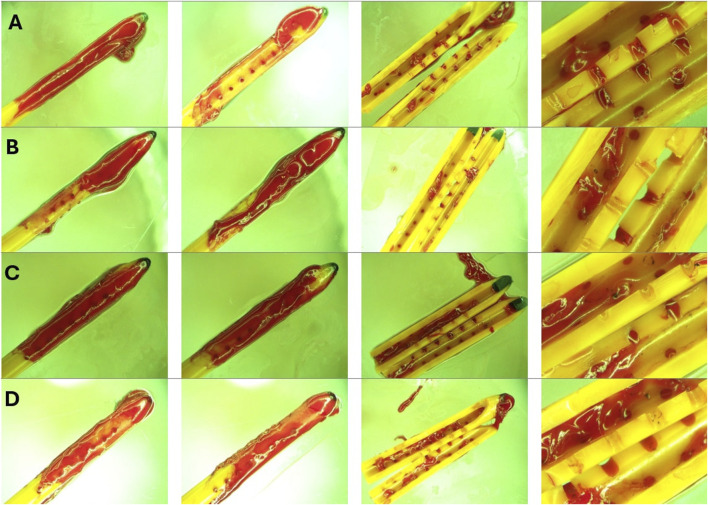
Endpoint view of external surfaces and the lumen of catheters in High Concentration (0.5%) Blood solution group. **(A)** Left to right: Front view, back view, lumen and zoomed in view of sample 1 after 30 cmH_2_O threshold was reached. **(B)** Left to right: Front view, back view, lumen and zoomed in view of sample 2 after 30 cmH_2_O threshold was reached. **(C)** Left to right: Front view, back view, lumen and zoomed in view of sample 3 after 30 cmH_2_O threshold was reached. **(D)** Left to right: Front view, back view, lumen and zoomed in view of sample 4 after 30 cmH_2_O threshold was reached. Note that a large bulbous blood clot was observed near the tip of all four catheters. Nevertheless, blood coagulation is observed everywhere across the length of the catheters. While extensive luminal obstruction was observed in Figures 10C,D, only a few blood clots were observed near the middle of the catheter in Figures 10B,C.

A detailed time-lapse of an antibiotic ventricular catheter during exposure to 0.7 $\frac{mL}{\min}$ bulk flow rate demonstrated the gradual process of clot formation (Figure 10). Clot formation became apparent everywhere, but especially near the lateral holes furthest from the catheter tip, forming a gradient of clot formation along the length of the catheter shortly after the initiation of flow. The non-uniform clot deposition continued until the pressure inside the chamber reached the 30 ${cmH}_{2}O$ threshold. Previous computational and benchtop publications, including our own, have demonstrated the non-uniform distribution of steady-state flow through the lateral holes of ventricular catheters such that the majority of flow occurs through the lateral holes furthest from the catheter tip (Roberts et al., 2024; Galarza et al., 2016; Weisenberg et al., 2018). Clot formation in Figure 10 appears to congregate around holes farthest from the catheter tip, where cylindrical and box computational models show elevated velocity fields around ventricular catheter holes. In Figure 11, we see a sleeve of coagulated blood that also infiltrates the holes. This may suggest that platelet and fibrin deposition in the holes is partially influenced by regional variations in flow velocity, which affects shear conditions. However, clotting is also observed on the catheter’s outer surface and lumen, where velocity fields differ significantly, indicating additional contributing factors. Pulsatile flow through the PETG bioreactors will influence the behavior of blood and blood-CSF mixtures due to their non-Newtonian properties, which affect viscosity and shear responses across varying velocity regions, including the lateral holes, lumen, and outer surface of the catheter. Future studies will examine how these properties impact flow behavior.

While variables such as the ventricular morphology and size impact CSF dynamics, the architecture of the shunt system also has a significant impact on drainage and obstruction formation (Galarza et al., 2016; Weisenberg et al., 2016; Galarza et al., 2015; Galarza et al., 2014). Despite similarities in overall appearance, EVDs and ventricular catheters are different in architectural dimensions, intended length of implantation, and clinical use. EVDs are generally utilized to manage acute hydrocephalus, while ventricular catheters are often a vital component of long-term CSF management in shunt-dependent patients. EVDs are commonly a part of clinical intervention for intracranial hemorrhage and thereby are at an elevated risk of thrombotic obstruction due to direct exposure to a mixture of blood and CSF mixture (Guimarães et al., 2021). This investigation demonstrated the drastic difference between the antibiotic-impregnated ventricular catheters and antibiotic-impregnated EVDs (Figure 5B), supporting our initial hypothesis that under similar conditions, EVDs have a significantly higher survival time compared to ventricular catheters.

Our recent publication demonstrated that architectural changes such as lumen diameter and lateral hole diameter had a statistically significant impact on the relative resistance of the catheters to flow *in vitro* (Madhavan et al., 2024). While identifying the relationship between shunting device architecture and survival time is beyond the scope of this investigation, a few notable differences between the EVDs and ventricular catheters were as follows: 1) EVDs included in this investigation had larger lumens and 2) EVDs included in this investigation had larger lateral holes compared to ventricular catheters (Figures 1C,D). Previous clinical studies reported a higher rate of occlusion in smaller EVDs (Fargen et al., 2016; Gilard et al., 2017). Despite statistically significant differences between EVDs and ventricular catheters, this study also demonstrated that in EVDs exposed to 5% Concentration blood solution, 30 ${cmH}_{2}O$ was reached, further highlighting the susceptibility of all shunt systems to thrombosis and the importance of future investigations into data-driven optimization of shunt systems.

Samples in High Concentration (0.5%) Blood Solution group, High Flow Rate (0.7 $\frac{mL}{\min}$ ) group, Barium-Impregnated group, and EVD-5% group were manually terminated when the 30 ${cmH}_{2}O$ threshold was reached. However, the time elapsed to reach the threshold was not constant between the groups. Additionally, a variety of pressure and flow patterns were observed in these groups. The variations in survival time, pressure, and flow patterns, and post-hoc distribution of blood clots might be associated with mechanistic processes that impacted the patency of the devices (Figures 6, 7). While the prior literature on the distribution and presence of blood clots on ventricular catheters and EVDs is limited, the blood clots observed in this investigation were similar to thrombosis observed in Central venous catheters (Citla Sridhar et al., 2020; Hadaway, 2005; Laguna et al., 2022).

Previous studies demonstrated the relevance and the advantages of Computational fluid dynamics for evaluating catheter-induced hemodynamic disturbances. Computational Fluid Dynamics was particularly impactful in the identification of wall shear stress, recirculation zones, and shear stress thrombosis potential (Piper et al., 2018; Wajihah and Sankar, 2023). Computational methods could be particularly useful in identifying the underlying mechanisms of shunt failure and play a pivotal role in the optimization process to improve the longevity of the shunt systems (Owen et al., 2020) (Figure 11). Particularly, we will look to future work by our group relaying flow parameters with biological responses, including that from blood.

**FIGURE 12:**
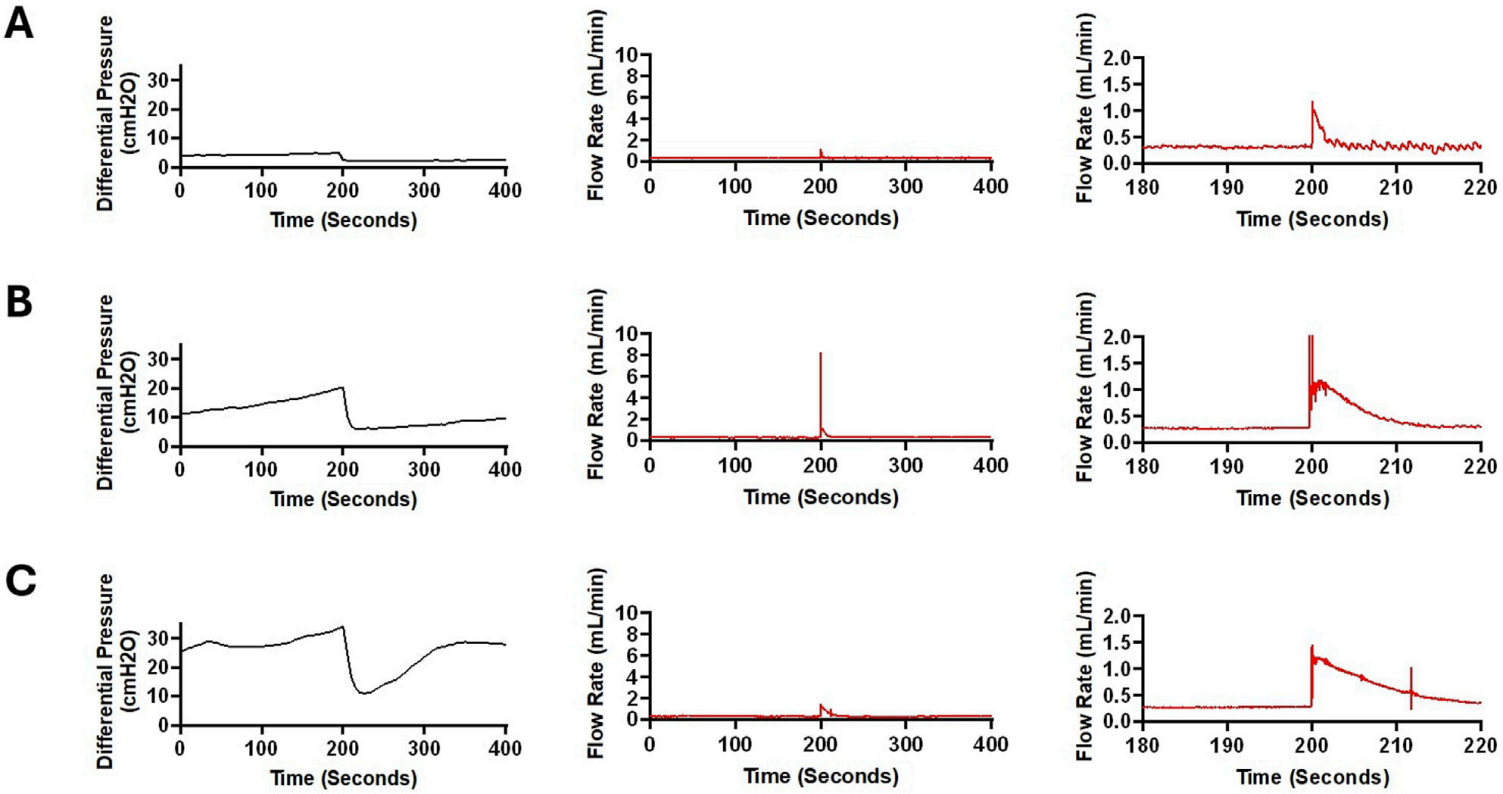
Sudden fluctuations in pressure and bulk flow rate in a sample from High Concentration (0.5%) Blood group. **(A)** In this figure, a 400-second segment of pressure and flow measurement recorded after around 5,200 s of pulsatile flow initiation is illustrated. A 2.448 cmH_2_O decrease in differential pressure was recorded within 5 s (Left). A sudden increase in flow was recorded with a peak amplitude of 1.19 mL/min (Middle). Note that in the zoomed in graph (Right), the flow appears more pulsatile after the peak recorded around the 200 s timepoint. This observation is consistent with the waveforms in Figure 6. The average flow rate between 0 and 100 s was 0.316 mL/min and the average flow rate between 300 and 400 s was 0.313 mL/min **(B)**. A 400-s segment of pressure and flow measurement recorded after around 7,200 s of pulsatile flow initiation is illustrated. A 14.144 cmH_2_O decrease in differential pressure was recorded within 15 s (Left). A precipitous increase in flow was recorded with a peak amplitude of 8.214 mL/min (Middle). The smooth transition of the waveform after the surge in flow rate is illustrated in the figure on the right. A slight difference in baseline flow rate was noted before and after the surge in flow such that the average flow rate between 0 and 100 s was 0.287 mL/min while the average flow rate between 300 and 400 s was 0.299 mL/min. **(C)**. A 400-second segment of pressure and flow measurement recorded after around 8,700 s of pulsatile flow initiation is illustrated. A 22.712 cmH_2_O decrease in differential pressure was recorded within 15 s (Left). Compared to Panels **(A,B)**, differential pressure recovered at a higher rate. An increase in flow was recorded with a peak amplitude of 1.456 mL/min (Middle). The gradual transition of the waveform after the surge in flow rate is illustrated (Right). The average flow rate between 0 and 100 s was 0.299 mL/min and the average flow rate between 300 and 400 s was 0.289 mL/min.

A previous benchtop study on the impact of 0.25%–1% blood solutions on the performance of shunt valves reported that higher concentrations of red blood cells and blood solution negatively impacted shunt valves or caused complete valve failure (Brydon et al., 1996). These results were consistent with our observations. However, the main objective of our study was to investigate the obstruction of proximal catheters and EVDs. Therefore, shunt valves were not included in our experimental setup. Nevertheless, pressure and flow measurements showed evidence of intermittent obstruction clearance. These episodes were presented as sudden surges in flow and short-term decreases in differential pressure (Figure 12). This preliminary evidence highlights the importance of including differential pressure valves in future investigations of shunt failure, as the dissociation of loosely held blood clots from proximal catheters could impact the operation of differential valves. The AIMS setup adapted for hemorrhage will be utilized in future work to investigate the impact of diluted blood solutions on shunt systems with differential pressure valves. The presented results also speak to the possibility that blood and other factors (e.g., tissue contact) may contribute to the clinical phenomena of intermittent obstruction. Finally, inflammatory mediators persistent after the resolution of the initial IVH may facilitate late clot formation that could impact the patency of the shunt system and result in shunt failure. The variances observed in the rate of clearing blood from CSF and patients’ age might also have an impact on shunt failure in clinical settings.

### Limitations

To our knowledge, this study is the first experimental investigation to record blood-induced shunt obstruction in real-time with direct cause-and-effect relationships due to blood concentration, bulk flow rate, and catheter type. Despite these promising results, the physiological and pathophysiological conditions of hydrocephalus are diverse and are difficult to recapitulate in a benchtop model (Devathasan et al., 2021). The exact mechanism of shunt obstruction is currently unknown but multifactorial, and in part dependent on the addition of blood in CSF (Zaranek et al., 2021). Several previous studies reported that the grade of hemorrhage is the strongest prognosticator of shunt revisions, and most complications occur shortly after the shunt insertion (Taylor and Peter, 2001; Dykes et al., 1989). However, most shunt obstructions in these studies occur over a more extended period of days and weeks, while catheters in our study failed within hours. These results suggest that the presented *in vitro* conditions accelerated the obstruction formation and shunt failure (Taylor and Peter, 2001). It is likely that fundamental differences between the *in vitro* and *in vivo* conditions, such as the volume, ventricles’ morphology, and compliance, impacted the rate of shunt obstruction. Furthermore, we hypothesize that under *in vivo* conditions, hemorrhage resolution processes have a significant impact on the patency and longevity of shunts in Patients with PHH. These processes might have been disrupted, inhibited, or were completely absent in the *in vitro* setup. Our previous investigation on the response of astrocytes to blood exposure reported a statistically significant increase in cellular attachment and glial fibrillary acidic protein (GFAP) expression in the samples that were exposed to blood (Zaranek et al., 2021). Future studies are warranted to investigate the survival time of ventricular catheters and EVDs in a model more representative of the *in vivo* conditions, composed of the presented PHH model and previously reported three-dimensional hydrogel scaffolds to introduce astrocytes by themselves or with other cell types as co-cultures (Harris et al., 2015).

One of the main limitations of this study is the small sample size due to the limited supply of commercial shunts for testing. Additionally, the catheters utilized in this experiment were past their shelf expiration date. While concerns regarding sterility were minimal in this investigation since whole porcine blood was utilized in short timelines, the efficacy and the impact of the antibiotic component past the expiration date were unknown, and the impact of potential antibiotic release over time was not studied. Future studies may utilize our novel high-throughput catheter manufacturing process to address the limited availability of catheters for research purposes.

The utilization of two synchronized, concurrent pump channels provided a reproducible and automated mode of hematoma introduction into the bioreactor chambers. However, the coagulability was likely impacted by heparin. Our investigation demonstrated that the coagulability of blood was at least partially restored, resulting in thrombosis and failure in a subset of catheters. However, we hypothesize that this experimental limitation could be addressed in future studies by utilizing blood drawn in real-time from an anesthetized donor as described in previous studies (MacLellan et al., 2008). This approach could also address another challenge in this experiment, the entrapment of small bubbles in the bioreactor chambers. While saline was degassed before the preparation of Solution A and Solution B, the preparation of homogeneous solutions introduced small bubbles. Most of the bubbles naturally escaped the solution beaker, but some of the bubbles were inevitably conducted into the chambers and remained near the top of the chamber, away from the catheter.

Our previous investigation reported the compatibility of PETG chambers with commercially available incubators to control ambient conditions (temperature, humidity, and ${CO}_{2}$ %) at a physiological range (Faryami et al., 2024). However, this investigation was conducted at room temperature because our preliminary experiment demonstrated that the humidity and the limited space inside the incubator interfered with the ability to record the obstruction formation in real time. To address this limitation, future experiments could forgo qualitative data collection and utilize commercially available incubators. This solution might be more suitable for samples that are exposed to 5% concentration blood solutions because of the low visibility of the catheter in these trials (Figure 8D). Alternatively, a custom-made incubator could be utilized to house the bioreactor chambers and the microscopes (Ross et al., 2022).

## Conclusion

A direct correlation was observed between blood concentration and pressurization rate, with shunts and EVDs exposed to higher blood concentrations consistently reaching the 30 cmH_2_O threshold, while those in low-concentration groups did not. In contrast, an inverse relationship was found between bulk flow rate and pressurization rate. Additionally, a significant difference was observed between ventricular catheters and EVDs. These findings underscore the role of clot formation in PHH shunt obstruction and suggest potential improvements in catheter design and clinical management strategies.

## Data Availability

The original contributions presented in the study are included in the article/Supplementary Material, further inquiries can be directed to the corresponding author.
