# Supplementary material for: Quantitative and qualitative investigation of shunt failure in an in vitro hemorrhagic hydrocephalus model
Source: Front Bioeng Biotechnol. 2025 Jul 1;13:1591952. doi: 10.3389/fbioe.2025.1591952 (PMC12259576; doi:10.3389/fbioe.2025.1591952)
Supplement: Supplementary file 5 [file DataSheet1.docx]

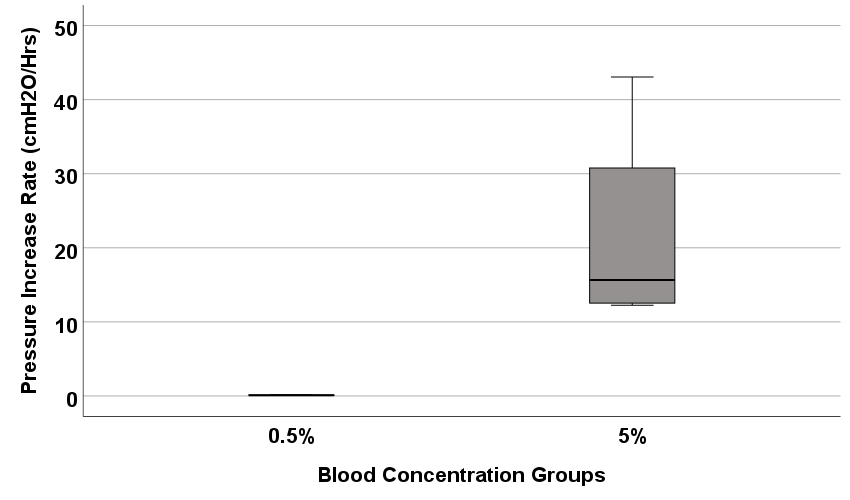


**S. Fig. 1. The investigation of differences in pressurization rate of EVDs in 0.5% and 5% blood concentration groups.**

**
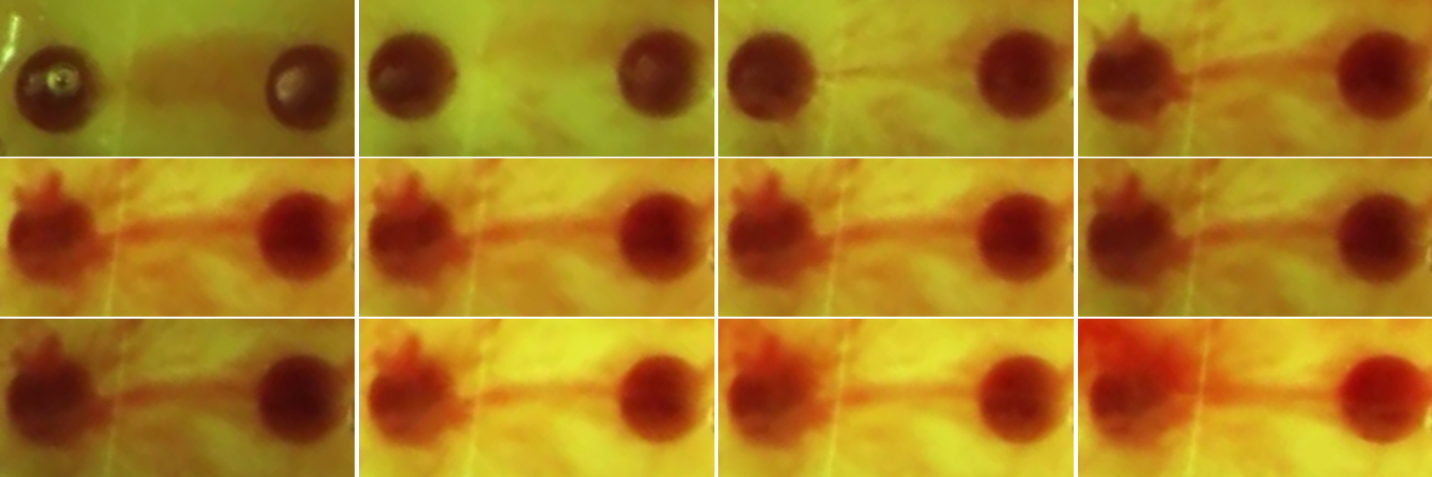
**

**S. Fig. 2. Zoomed in frames from S. Vid 2 illustrating the formation of a fibrotic bridge across the lateral holes of the catheters.** The frames demonstrate the expansion of a fibrotic bridge between the second (left) and the third (right) holes from the tip of the antibiotic ventricular catheter. It appeared that the fibrotic bridge expanded from the third lateral hole towards the second lateral hole.
